# Kidney disease in Uganda: a community based study

**DOI:** 10.1186/s12882-017-0521-x

**Published:** 2017-04-03

**Authors:** Robert Kalyesubula, Joaniter I. Nankabirwa, Isaac Ssinabulya, Trishul Siddharthan, James Kayima, Jane Nakibuuka, Robert A. Salata, Charles Mondo, Moses R. Kamya, Donald Hricik

**Affiliations:** 1 School of Medicine, Makerere University College of Health Sciences, Kampala, Uganda; 2 Mulago National Referral Hospital, Kampala, Uganda; 3 Uganda Heart Institute, Kampala, Uganda; 4 Johns Hopkins University, Baltimore, USA; 5 Case Western Reserve University, Ohio, USA; 6 Departments of Medicine and Physiology, Makerere University, P.O. Box 7072, Kampala, Uganda

**Keywords:** Chronic kidney disease, Risk factors, Uganda, sub-Saharan Africa

## Abstract

**Background:**

Chronic kidney disease (CKD) is a major cause of morbidity and mortality in Sub-Saharan Africa (SSA). The majority of studies on CKD in SSA have been conducted among HIV-infected populations and mainly from large health facilities. We determined the prevalence of CKD and its predictors among populations in communities in central Uganda.

**Methods:**

A cross-sectional study was conducted in Wakiso district using multi-stage sampling. Data was collected on age, sex, socio-economic status, history of alcohol intake, diabetes mellitus, hypertension and smoking. Measurement of blood pressure, weight and height to determine body mass index (BMI) and investigations including HIV testing, fasting blood sugar, creatinine and urinalysis were conducted. Logistic regression was used to estimate the strength of the association between variables and the presence of CKD estimated using the Cockcroft Gault formula.

**Results:**

A total of 955 participants aged 18–87 years were enrolled into the study. The median age was 31 years (Interquartile range 24–42) and majority (67%) were female. Up to 21.4% (204/955) had abnormal renal function with CKD stage 1 in 6.2% (59/955), stage 2 in 12.7% (121/955), stage 3 in 2.4% (23/955), CKD stage 4 in 0% and CKD stage 5 in 0.1% (1/995). Female gender OR 1.8 (95% Confidence Interval [CI] 1.2–2.8), age >30 years OR 2.2(95% CI 1.2–3.8) and high social economic status OR 2.1 (95% CI 1.3–3.6) were associated with increased risk of CKD while BMI > 25Kg/m^2^ was protective against CKD OR 0.1 (95% CI 0.04–0.2). Traditional risk factors such as HIV-infection, diabetes mellitus, smoking and alcohol intake were not found to be significantly associated with CKD.

**Conclusion:**

We found a high prevalence of kidney disease in central Uganda. Interestingly the traditional risk factors associated with CKD previously documented, were not associated with CKD.

## Background

The global burden of non-communicable diseases (NCDs) continues to increase. The World Health Organization projects that NCDs will account for 46% of mortality in sub-Saharan Africa (SSA) by 2030 [1]. Currently, SSA has a dual burden of both infectious and non-infectious diseases. Chronic kidney disease (CKD) plays a major role as a cause and a consequence of other NCDs. A recent systematic review estimates the current prevalence of CKD in sub-Saharan Africa (SSA) at 13.9% (95% Confidence Interval [CI] 12.2–15.7) [2]. It is also estimated that by 2030, 70% of worldwide end-stage renal disease (ESRD) will be in low income countries like those in SSA [3]. In many countries the majority of CKD-related studies have focused on HIV-infected patients [4]. In Uganda, a plurality of studies conducted in the recent past has focused on HIV-infected patients with little data available on non HIV-infected people [5–9]. This study involved participants with and without HIV in rural and semi-urban communities of Uganda.

There are many challenges concerning managing kidney diseases in SSA. First, we currently lack data on the major risk factors for chronic kidney disease in SSA due to lack of renal registries and meticulous longitudinal studies [10, 11]. The second challenge is that the majority of patients have no symptoms in the early stages of kidney disease and only present with advanced disease to health care facilities. In a recent study from a national referral hospital from Uganda, up to 51% (*n* = 111) of patients attending the outpatient nephrology care clinic had ESRD [12]. This is largely because the risk factors that could trigger a high index of suspicion for kidney disease in most of SSA have not been well established [11]. Once a patient develops end stage kidney disease there is need for renal replacement therapy irrespective of the primary cause. Early diagnosis and treatment on the other hand has been shown to delay or even reverse CKD but this is largely driven by screening for known risk factors and treating them [13, 14].

We determined the prevalence for CKD and associated factors in both rural and urban based community settings of Wakiso, Uganda using a cross-sectional design. We also compared the prevalence generated using the different glomerular estimation formulae including the Chronic Kidney Disease -Epidemiology Consortium equation (CKD-Epi), the Cockcroft Gault (CG) and the Modification of Diet in Renal Disease (MDRD) to establish a baseline differences between the formulae.

## Methods

**Fig. 1:**
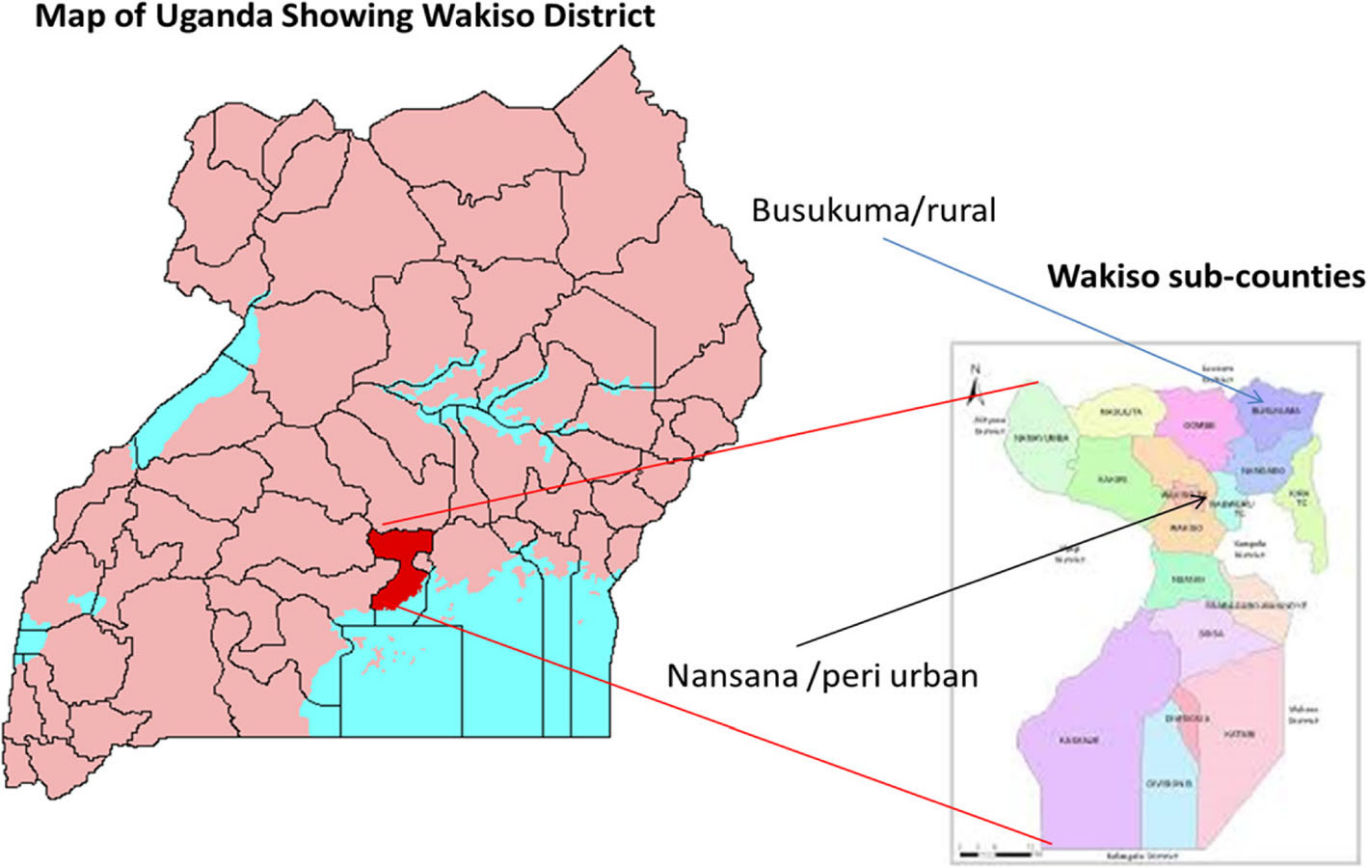
Map of Uganda Showing urban and rural areas from Wakiso district. This map was adopted and modified by Dr Robert Kalyesubula from wikimedia

Between August 2012 and August 2013, MEPI-CVD (Medical Education Partnership Initiative on Cardiovascular Diseases) conducted a cross-sectional community based survey in an urban and a rural sub-county in Wakiso district to determine the prevalence and risk factors for cardiovascular disease [15]. The detailed methods of the MEPI-CVD survey have been described elsewhere [15, 16]. All the sub-counties in Wakiso district were stratified into rural and urban and then one sub-county was chosen from each stratum by simple random sampling. One urban sub-County (Nansana Town Council) was randomly selected out of 5 urban sub-counties and one rural sub-County (Busukuma) was randomly selected out of 13 rural sub-counties in Wakiso district (see Fig. 1. Study Area: Map showing rural area of Busukuma and periurban area of Nansana, Wakiso District, Uganda. Caption: This image was modified from Wikimedia Commons for maps by Dr. Robert Kalyesubula). All households and other key features in the selected sub-counties were mapped and enumerated to generate a sampling frame for the survey. Household locations were mapped using hand-held global positioning system (GPS) receivers. A total of 5,420 households was selected to participate in the survey using a list randomly generated from the household enumeration database. In each of the selected households, one adult (18 years or older) was selected to participate in the survey. Households without an adult were excluded leaving a total of 4,952 participants in the study. Following informed consent, a structured standard questionnaire adopted from WHO STEPS was administered to the household respondents. The questionnaire was administered through face-to-face personal interviews in a research clinic, ensuring a setting that provided maximum privacy to conduct the interview. The data collected included: demographics (age, sex, and address), diet, tobacco and alcohol consumption and medical history, as well as smoke exposure, socio-economic status and family history of NCDs. The researchers also undertook physical measurements for blood pressure, height, weight to obtain the BMI, waist circumference and hip circumference to obtain the waist-hip ratio following standard protocols. Blood samples were obtained via venous phlebotomy for investigations including; HIV, fasting blood sugar and full blood counts. Urine analysis was done using a dipstick. All participants from the primary MEPI-CVD linked study with complete data including urine protein were eligible for the CKD study.

During the MEPI-CVD survey, blood samples were stored as plain serum in microvials in a negative 80 °C freezer at the MBN Clinical Laboratory in Kampala, Uganda. The MBN Clinical laboratory is an internationally accredited laboratory with quality standards for both real time and retrospective specimen analysis. We retrieved the stored samples for the randomly selected 1000 participants who met the inclusion criteria and tested their creatinine levels using the Cobas Auto Analyzer (Roche Diagnostics, North America) with a standard reference range of; 66 -106umol/L. Of the 1000 participants 955 had complete samples. The creatinine level measured for each of the participants was used to estimate the glomerular filtration rate using the Cockcroft-Gault formula adjusted for BMI in accordance with previous studies performed in Uganda [6].

### Statistical analysis

To determine the prevalence of kidney disease, we used the proportion of patients with proteinuria of > 1+ with an estimated glomerular filtration rate of less than 90mls/min/1.73 m^2^ and divided this by the total number of patients recruited in the study. Proteinuria was graded as: negative (less than 10 mg per dL), trace (10 to 20 mg per dL), 1+ (30 mg per dL), 2+ (100 mg per dL), 3+ (300 mg per dL) or 4+ (1,000 mg per dL) [17]. Chronic kidney disease was further staged according severity based on the National Kidney Foundation guidelines [18]. We defined kidney disease as a creatinine clearance of less than 90mls/min/1.73 m2 and chronic kidney disease as a creatinine clearance of less than 60mls/min/1.73 m2. Z-scores were generated to compare the proportions at each severity level using the CKD-Epi values as reference [19]. The four categories of the Socio-economic Score (SES) were derived from conducting Principle Component Analysis (PCA) on a statistical software using variables relating to household infrastructure and property ownership. Logistic regression was used to identify factors associated with having kidney disease and odds ratios with their 95% confidence intervals as the measures of association. We adhered to the STROBE guidelines/methodology for reporting our findings.

### Ethics

Ethical approval was obtained from Makerere University School of Medicine Research and Ethics Committee as well as the Uganda National Council for Science and Technology (UNCST). Patients provided informed consent for the parent study (MEPI-CVD study) and future use of the samples which were stored. Administrative permission to use the stored samples was also obtained from the Principal Investigator of the MEPI-CVD study.

## Results

### Characteristics of participants

Of 1000 patients screened from the MEPI-CVD study, 955 (96%) had complete data to determine the prevalence and associated factors for CKD. The remaining 45 participants had incomplete samples for measurement of creatinine but did not differ significantly from those included in the study. We additionally compared the demographics of the 955 participants included for the CKD study with the 3,997 excluded from the original study and found no differences in demographic variables.

Of the 955 participants, the majority (67%, *n* = 640) were female and more than two-thirds (73%, *n* = 696) were peri-urban residents. The median age of the participants was 31 years (IQR, 24–42). A total of 93 (9.7%) participants were HIV-infected, while one in four (25%, *n* = 239) were hypertensive-defined as being on drugs for hypertension or having a systolic blood pressure ≥ 140 mmHg and/or diastolic blood pressure ≥ 90 mmHg at three consecutive measurements. The majority of participants had never smoked (90%, *n* = 862) nor used alcohol (88%, *n* = 841). A summary of selected characteristics of the participants by gender is presented in Table 1. The rural and peri-urban communities significantly differed on all demographic factors except HIV- infection status and presence of proteinuria. Participants from peri-urban communities were more likely to be younger, poorer, and normotensive with higher BMI (see Table 2).

**Table 1:** Baseline characteristics of study participants from Wakiso, Uganda by gender

Characteristic	Total *N* = 955	Males (*N* = 315)	Female (*N* = 640)
Median age (IQR)	31 (24–42)	30 (24–40)	32 (25–43)
Age group			
18–29	410 (42.9)	151 (47.9)	259 (40.5)
30–39	261 (27.3)	78 (24.8)	183 (28.6)
40–49	136 (14.2)	48 (15.2)	88 (13.7)
50–59	73 (7.6)	17 (5.4)	56 (8.8)
60+	75 (7.9)	21 (6.7)	54 (8.4)
Sub-county			
Nansana Town Council	696 (72.9)	213 (67.6)	483 (75.5)
Busukuma	259 (27.1)	102 (32.4)	157 (24.5)
SES^a^			
Poorest	226 (23.7)	75 (23.8)	151 (23.6)
Poor	225 (23.6)	85 (26.9)	140 (21.9)
Less poor	261 (27.3)	80 (25.4)	181 (28.3)
Least poor	243 (25.4)	75 (23.8)	168 (26.2)
Median Weight (IQR)	61 (53–71)	61 (56–69)	60 (52–72)
BMI groups			
Underweight	52 (5.5)	24 (7.6)	28 (4.4)
Normal	544 (56.9)	230 (73.0)	314 (49.1)
Overweight	231 (24.2)	49 (15.6)	182 (28.4)
Obese	128 (13.4)	12 (3.8)	116 (18.1)
HIV-Infection			
No	862 (90.3)	289 (91.8)	573 (89.5)
Yes	93 (9.7)	26 (8.2)	67 (10.5)
Diabetic n (%)			
Normal	733 (76.8)	271 (86.0)	462 (72.2)
Impaired tolerance	173 (18.1)	38 (12.1)	135 (21.1)
Diabetic	49 (5.1)	6 (1.9)	43 (6.7)
Hypertension n (%)			
Normal	716 (75.0)	235 (74.6)	481 (75.2)
Hypertensive	239 (25.0)	80 (25.4)	159 (24.8)
Smoking, n (%)			
Never smoked	862 (90.3)	259 (82.2)	603 (94.2)
Previously smoker	39 (4.1)	17 (5.4)	22 (3.4)
Current smoker	54 (5.6)	39 (12.4)	15 (2.3)
Alcohol use n (%)			
None	841 (88.1)	254 (80.6)	587 (91.7)
Mild	33 (3.5)	16 (5.1)	17 (2.6)
Moderate	52 (5.4)	25 (7.9)	27 (4.2)
Heavy	29 (3.0)	20 (6.4)	9 (1.4)
Mean serum creatinine (SD)	0.66 (0.18)	0.75 (0.2)	0.62 (0.2)
CCr (mls/min/1.73 m2)median (IQR)			
CG	124 (101–158)	124 (103–157)	124 (101–160)
MDRD	140 (116–177)	149 (123–188)	136 (112–170)
CKD-Epi	138 (122–152)	141 (128–155)	136 (119–150)
Proteinuria (urine protein ≥ 1+)			
No	952 (99.7)	314 (99.7)	638 (99.7)
Yes	3 (0.3)	1 (0.3)	2 (0.3)

*Abbreviations: BMI* Body mass index*, CG* Cockroft Gault*, CCr* creatinine clearance*, CKD-EPI* Chronic Kidney Disease Epidemiology Consortium equation*, IQR* Interquartile range*, MDRD 4-*variable Modification of Diet in Renal Disease equation*,* SES social economic score

^a^Socio-economic Score (SES) derived from conducting Principle Component Analysis (PCA) on a statistical software using variables relating to household infrastructure and property ownership

**Table 2:** Baseline characteristics of study participants from Wakiso, Uganda by sub-county

Characteristic	Nansana (*N* = 696)	Busukuma (*N* = 259)	*p*-value
Median age (IQR)	30 (24–38)	38 (27–50)	<0.001
Age group			
18–29	335 (48.1)	75 (28.9)	<0.001
30–39	202 (29.0)	59 (22.8)	0.056
40–49	80 (11.5)	56 (21.6)	<0.001
50–59	44 (6.3)	29 (11.2)	<0.011
60+	35 (5.0)	40 (15.4)	<0.001
Sex			
Male	213 (30.6)	102 (39.4)	<0.001
Female	483 (69.4)	157 (60.6)	
SES			
Poorest	205 (29.4)	21 (8.1)	<0.001
Poor	192 (27.6)	33 (12.8)	<0.001
Less poor	126 (18.1)	135 (52.1)	<0.001
Least poor	173 (24.9)	70 (27.0)	0.508
Median Weight (IQR)	62 (55–72)	58 (50–66)	<0.001
BMI groups			
Underweight	31 (4.5)	21 (8.1)	0.029
Normal	365 (52.4)	179 (69.1)	<0.001
Overweight	186 (26.7)	45 (17.4)	0.003
Obese	114 (16.4)	14 (5.4)	<0.001
HIV-Infection			
No	622 (89.4)	240 (92.7)	0.126
Yes	74 (10.6)	19 (7.3)	
Diabetic n (%)			
Normal	516 (74.2)	217 (83.8)	0.002
Impaired tolerance	139 (19.9)	34 (13.1)	0.015
Diabetic	41 (5.9)	8 (3.1)	0.082
Hypertension n (%)			
Normal	535 (76.9)	181 (69.9)	0.026
Hypertensive	161 (23.1)	78 (30.1)	
Smoking, n (%)			
Never smoked	631 (90.7)	231 (89.2)	<0.001
Previously smoker	31 (4.4)	8 (3.1)	0.365
Current smoker	34 (4.9)	20 (7.7)	0.096
Alcohol use n (%)			
None	622 (89.4)	219 (84.6)	0.042
Mild	23 (3.3)	10 (3.9)	0.652
Moderate	31 (4.5)	21 (8.1)	0.029
Heavy	20 (2.9)	9 (3.5)	0.632
Mean serum creatinine (SD)	0.65 (0.54–0.76)	0.67 (0.53–0.81)	0.297
CG	130 (87.7–161.5)	112 (88.9–142.5)	<0.001
MDRD	141 (117.4–177.5)	139 (108.5–174.7)	0.859
CKD-Epi	139 (125.1–153.1)	132 (114.9–147.1)	<0.001
Proteinuria (urine protein ≥ 1+)			
No	693 (99.6)	259	0.308
Yes	3 (0.4)	0	

Socio-economic Score (SES) derived from conducting Principle Component Analysis (PCA) on a statistical software using variables relating to household infrastructure and property ownership

*Abbreviations: BMI* Body mass index, *CG* Cockroft Gault, *CCr* creatinine clearance, *CKD-EPI* Chronic Kidney Disease Epidemiology Consortium equation, *IQR* Interquartile range, *MDRD* 4-variable Modification of Diet in Renal Disease equation, *SES* social economic score

### Prevalence of kidney disease

**Fig. 2:**
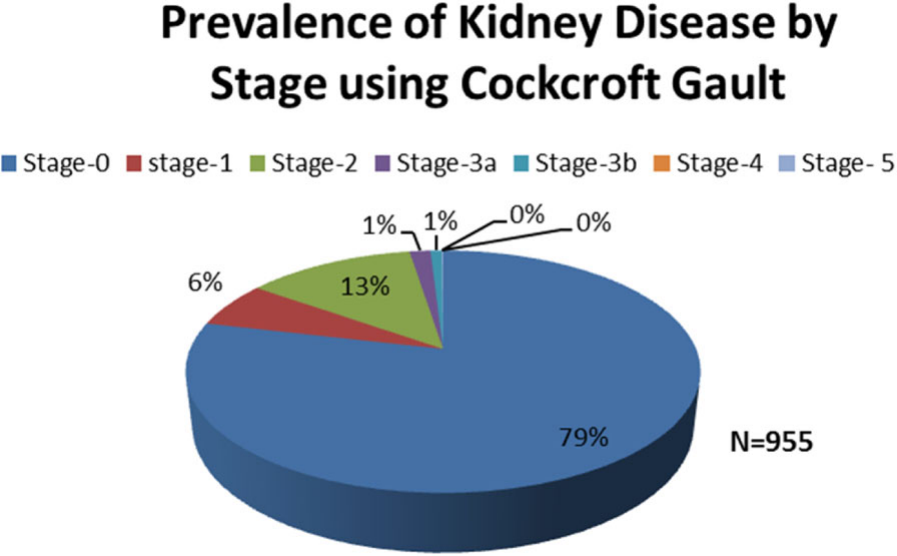
Prevalence of kidney disease by stage

The prevalence of kidney disease in this study population was 15.2% (*n* = 145) using the Cockcroft Gault formula. The prevalence according to CKD stages were; CKD stage 1 in 6.2% (*n* = 59), stage 2 in 12.7% (*n* = 121), stage 3 in 2.4% (*n* = 23), stage 4 in 0% (*n* = 0) and stage 5 (end stage kidney disease) in 0.1% (*n* = 1). A total of 6.2% (59) participants had normal glomerular filtration rates but with urinary abnormalities. More than a fifth (21.4%, *n* = 204) of the participants had renal abnormalities of creatinine and urinalysis detected (see Fig. 2). Of those with urine abnormalities 3 (0.3%) had proteinuria and 55 (5.6%) participants had hematuria.

### Comparison of the prevalence of stages of CKD using the different formulae for estimation of glomerular filtration rate

Sensitivity analysis was done using different formulae. The average glomerular filtration rates did not differ (see Table 3). Using the three internationally recognized estimated glomerular filtration rate (eGFR) formulae namely: Chronic Kidney Disease -Epidemiology Consortium equation(CKD-Epi), Modification of Diet in Renal Disease (MDRD) and the Cockcroft Gault (CG); more than a fifth of participants had kidney abnormalities. The prevalence for stage 1 kidney disease was similar using the three formulae. Significant differences in prevalence were noted for stage 2 kidney disease with CKD-Epi detecting 2.9% (*n* = 28), CG at 12.7% (*n* = 121) and MDRD showing 5.9% (*n* = 57) (*p* < 0.001). For advanced kidney disease stage 4 and 5; there were no patients with CKD identified by the CKD-Epi or MDRD formulae but one patient (0.1%) was identified to have ESRD with the CG formula (see Table 4).

**Table 3:** Glumerular filtration rates by different formulas

eGFR	Median, IQR (males)	Median, IQR (Females)	Median, IQR (Overall)
eGFR-CG	124.1 (102.6–157.2)	123.6 (101.1–159.5)	123.9 (101.4–158.0)
eGF- MDRD	148.9 (122.6–187.9)	135.6 (111.7–170.1)	140.5 (115.8–176.6)
eGFR- CKD-Epi	140.8 (127.5–155.0)	135.8 (119.4–149.6)	137.6 (121.9–152.1)

*eGFR* estimated glomerular filtration rate, all measurements in mls/min/1.73 m2, *CG* Cockroft Gault, *MDRD* 4-variable Modification of Diet in Renal Disease equation, *CKD-EPI* Chronic Kidney Disease Epidemiology Consortium equation

**Table 4:** Chronic Kidney Disease stages according to the National Kidney Foundation, using Cockroft Gault and Modification of Diet in Renal Disease Glomerular Filtration Rate estimations compared with CKD-Epi

	GFR estimation	CKD-Epi *n* (%) *N* = 955	CG est *n* (%) *N* = 955	p (CG vs CKD –Epi)	MDRD n (%)	p (MDRD vs CKD –Epi)
0	≥90, without urine dipstick abnormality	861 (90.2)	751 (78.6)	<0.0001	834 (87.3)	0.045
1	≥ 90, with urine dipstick abnormality	64 (6.7)	59 (6.2)	0.656	62 (6.5)	0.860
2	60–89, with urine dipstick abnormality	28 (2.9)	121 (12.7)	<0.0001	57 (5.9)	0.001
3a	45–59.9, with or without urine dipstick abnormality	1 (0.1)	15 (1.6)	0.0004	1 (0.1)	1.000
3b	30–44.9, with or without urine dipstick abnormality	1 (0.1)	8 (0.8)	0.022	1 (0.1)	1.000
4	15–29.9, with or without urine dipstick abnormality	0	0	1.000	0	1.000
5	< 15, with or without urine dipstick abnormality	0	1 (0.1)	0.328	0	1.000

*Abbreviations: CG* Cockroft Gault*, CKD-Epi* Chronic Kidney Disease Epidemiology Consortium equation*, e-GFR* estimated glomerular filtration rate*, MDRD* 4-variable Modification of Diet in Renal Disease equation

### Risk factors for kidney disease

Being female OR 1.8 (95% CI 1.2–2.8), age over 30 years OR 2.2 (95% CI 1.2–3.8) and a high social economic status (least poor) OR 2.1 (95% CI 1.3–3.6) were associated with kidney disease. The odds for age increased exponentially for higher age groups. Body mass index (BMI) above 25 Kg/m^2^ was protective against CKD OR 0.1 (95% CI 0.04–0.2).

On bivariate analysis, participants from rural Busukuma were three times OR 3.2 (95% CI 2.2–4.5) more likely to have kidney disease compared to their peri-urban counterparts from Nansana. However, this association diminished on adjusting for confounders including age and social economic status. Similarly, on bivariate analysis, participants with hypertension were 2 times more likely to have kidney disease but this was largely confounded by age and body mass index (see Table 5). The traditional risk factors of HIV-infection, diabetes mellitus, smoking and alcohol intake were not significantly associated with kidney disease in this study (see Table 5).

**Table 5:** Association between kidney disease (Cockcroft-Gault formula) and different predictor variables

Characteristic	n/N (%)	Unadjusted OR (95% CI)	*p*-value	Adjusted OR (95% CI)	*p*-value
Sub-county					
Nansana	74/696 (10.6)	1		1	
Busukuma	71/259 (27.4)	3.2 (2.2–4.5)	<0.001	1.5 (0.99–2.4)	0.053
Sex					
Male	41/315 (13.0)	1		1	
Female	104/640 (16.2)	1.3 (0.2–1.9)	0.191	1.8 (1.2–2.8)	0.010
Age group					
18–29	28/410 (6.8)	1		1	
30–39	27/261 (10.3)	1.6 (0.9–2.7)	0.108	2.2 (1.2–3.8)	0.009
40–49	32/136 (23.5)	4.2 (2.4–7.3)	<0.001	4.3 (1.2–3.8)	<0.001
50–59	17/73 (23.3)	4.1 (2.1–8.1)	<0.001	5.5 (2.4–7.8)	<0.001
60+	41/75 (54.7)	16.5 (9.1–29.8)	<0.001	17 (8.4–35.4)	<0.001
SES					
Poorest	25/226 (11.1)	1			
Poor	21/225 (9.3)	0.8 (0.4–1.5)	0.545		
Less poor	55/261 (21.1)	2.1 (1.3–3.6)	0.003		
Least poor	44/243 (18.1)	1.8 (1.1–3.0)	0.033		
BMI groups					
Underweight	21/52 (40.4)	1		1	
Normal	105/544 (19.3)	0.3 (0.2–0.6)	0.001	0.4 (0.2–0.8)	0.008
Overweight	18/231 (7.8)	0.1 (0.05–0.3)	<0.001	0.1 (0.04–0.2)	<0.001
Obese	1/128 (0.8)	0.01 (0.002–0.09)	<0.001	0.01 (0.001–0.1)	<0.001
HIV-Infection					
No	130/862 (15.1)	1			
Yes	15/93 (16.1)	1.1 (0.6–1.9)	0.789		
Diabetic					
Normal	106/733 (14.5)	1		1	
Impaired tolerance	29/173 (16.8)	1.2 (0.8–1.9)	0.445	1.2 (0.7–2.0)	0.544
Diabetic	10/49 (20.4)	1.5 (0.7–3.1)	0.260	2.4 (0.98–5.8)	0.055
Hypertension					
Normal	92/716 (12.9)	1		1	
Hypertensive	53/239 (22.2)	1.9 (1.3–2.8)	0.001	1.2 (0.7–1.9)	0.474
Smoking					
Never smoked	133/862 (15.4)	1			
Previously smoker	3/39 (7.7)	0.5 (0.1–1.5)	0.198		
Current smoker	9/54 (16.7)	1.1 (0.5–2.3)	0.808		
Alcohol use					
None	132/841 (15.7)	1			
Mild	2/33 (6.1)	0.3 (0.1–1.5)	0.150		
Moderate	8/52 (15.4)	0.9 (0.4–2.1)	0.952		
Heavy	3/29 (10.3)	0.6 (0.2–2.1)	0.438		

Age-group confounds HT, BMI confounds HT

We also run an analysis using the CKD-Epi and MDRD for CKD associated factors (data not shown). Female sex, age above 30 years, and socio-economic status were found to be associated with CKD using the MDRD and CG formulae. Age above 30 years and hypertension were found to be associated with CKD using the CKD-Epi formula. Diabetes mellitus was not consistently identified as a risk factor for kidney disease with MDRD identifying it as a significant factor while CG and CKD-Epi did not.

## Discussion

We found a high prevalence of kidney disease at 15.2%, CKD of 2.5% with ESRD in 0.1% in Wakiso district, Uganda. The high prevalence of kidney disease in rural and peri-urban areas highlights the current burden of NCDs. High prevalence of kidney disease has been identified in other areas of SSA; in Tanzania the prevalence of CKD was found to be 7.0% overall with 15.2% in the urban population while a systematic review estimates an overall prevalence of CKD in SSA at 13.9% [2, 10]. These findings have far reaching consequences to a developing country of 34.9 million people [20] with few trained specialist and few dialysis centres to support the increased burden of kidney disease [21]. The cost of caring for patients with ESRD is extremely high. Hemodialysis which is one of the available means for management of ESRD in SSA has been estimated to cost between 7,000 and 55,000 US dollars per patient per year depending on whether the facility is private or government supported [21–23]. For a perspective, the average household (average of 5 persons) monthly income for Ugandans is estimated at a meager 78 US dollars per month with an average monthly expenditure of 12 US dollars on healthcare [24, 25].

There was a significant difference in the prevalence and stages of chronic kidney disease when different formulae were used to estimate the glomerular filtration rate. Similar findings have been noted in other studies. A study by Wyatt [26] found that CG over estimates the rate of CKD while Kaze noted different rates of CKD ranging from 4.4 to 8.8% depending on which formula was used [27]. The CKD-Epi has been found to be more accurate in patients with near normal kidney function [28]. However, highest correlation in clinical practice was found with CG when corrected for low body mass [29]. The equations used to determine the estimated glomerular filtration rate (eGFR) have not been validated among populations in SSA [30, 31]. Thus, it is still difficult to determine the optimal eGFR formula to use in establishing the prevalence and risk factors for CKD particularly in resource-limited settings. Variability in formulae for diagnosing CKD has far reaching implications as patients may be misclassified and given the wrong diagnostic stage which may lead to anxiety and unnecessary treatments if the diagnostic stage is worse than the true GFR. Conversely, patients may miss treatment if they are given a more favourable diagnostic stage than their true GFR. It is therefore critical that a true GFR is determined for each patient. Resolving this issue will require larger population-based studies in SSA to determine the true GFR formula for this region.

Age (above thirty years), female gender, and high social economic status were associated with increased risk of kidney disease. This has been found by other studies from Africa [2, 27]. In contrast, low socioeconomic status has been associated with both development and progression of CKD in Europe and United States of America [25, 32]. This may be attributed to environmental and lifestyle factors which differ across the different regions [33]. In general, the prevalence of CKD is greater among women than in men across all ages from both developed countries and SSA [34]. The difference in CKD prevalence between males and females could be explained by the fact that women have less muscle mass and there are differences in the glomerular structure and hormone metabolism which may play a role in this gender disparity [35]. We did not find a significant difference in prevalence between peri-urban Nansana Town Council and rural Busukuma. This is in contrast to the study in neighbouring Tanzania where rural communities had a low prevalence at 2.0% compared with the urban in 15.7% [10]. The complicated association between age, poverty and BMI in urban and rural areas may have had an impact on the results between these two communities.

Diabetes mellitus, smoking and alcohol intake were not key factors associated with kidney disease in this study population, contrary to previous findings. This may be due the fact that the majority of participants did not report use of alcohol or smoking. In a recently completed national survey for diabetes in Uganda, only 1.4% (95% CI 0.9–1.9%, *N* = 3689) participants had diabetes mellitus [36]. The low prevalence of these well known risk factors for CKD in our participants makes any comment of their impact difficult to ascertain. In a study by Stanifer et al., half of the cases of CKD (49.1%) were not associated with any of the measured risk factors of hypertension, diabetes, or HIV-infection [10]. In developed countries and worldwide, the majority of kidney disease is due to diabetes mellitus followed by hypertension [37]. It is thus still unclear as to what may be driving the high levels of kidney disease in the rural and semi-urban areas of SSA. Future research needs to look into the peculiar drivers of CKD in SSA. Hypertension and diabetes mellitus can easily be diagnosed even at the lower facility levels. But if there are other unknown causes such as glomerular (especially post-infectious) and interstitial renal diseases driving the epidemic of kidney disease in Uganda, these need to be urgently identified so that appropriate steps to prevent progression to end-stage renal disease can be effected. Treatment of ESRD is not clinically and economically feasible for most low- and middle-income countries [38].

Our study had several strengths. This was a large community based survey, an attribute that is likely to give a proxy estimate of the burden of kidney disease in Uganda. In addition, our study population was both rural and semi-urban and therefore in a true epidemiological transition. The results of our study can therefore be generalized to many populations on the African continent that are in a similar transition. We also compared the three e-GFR estimation methods and highlighted the significant differences that exist between them which may have far reaching consequences to patient care.

However, our study also had a few limitations. The primary study was a survey, cross sectional in nature and some data on key parameters for establishing chronic kidney disease like the abdominal ultrasound scan were missing. We used proteinuria as a surrogate marker well aware that it may be affected by other causes like urinary tract infections among others. Furthermore, proteinuria rather than microalbuminuria was used for this study. In addition, we were also not able to determine whether the kidney dysfunction was acute or chronic because we did not have repeat (>3 month) creatinine. Nevertheless, we believe that this study will create awareness on the magnitude of kidney disease in Uganda and hopefully stimulate further research into the underlying etiology and set in motion ways to prevent it.

## Conclusion

We found a high prevalence of kidney disease in Uganda which could not be explained by the traditional causes of CKD and noted significant levels of disagreement in the formulae used to estimate glomerular filtration rates. Further studies are needed to characterize the drivers of kidney disease beyond diabetes and hypertension as the prevalence of these diseases continues to rise.

## Abbreviations

BMI: Body mass index
CG: Cockcroft Gault
CKD: Chronic kidney disease
CKD-Epi: Chronic kidney disease -epidemiology consortium equation
e-GFR: Estimated glomerular filtration rate
ESRD: End-stage renal disease
GFR: Glomerular filtration rate
MDRD: Modification of diet in renal disease
MEPI-CVD: Medical education partnership initiative on cardiovascular diseases
NCDs: Non communicable diseases
PCA: Principle component analysis
SES: Socio-economic Score
SSA: Sub-Saharan Africa
UNCST: Uganda National Council for Science and Technology (UNCST)
